# Glycolate oxidase-dependent H_2_O_2_ production regulates IAA biosynthesis in rice

**DOI:** 10.1186/s12870-021-03112-4

**Published:** 2021-07-06

**Authors:** Xiangyang Li, Mengmeng Liao, Jiayu Huang, Zheng Xu, Zhanqiao Lin, Nenghui Ye, Zhisheng Zhang, Xinxiang Peng

**Affiliations:** 1 State Key Laboratory for Conservation and Utilization of Subtropical Agro-Bioresources, College of Life Sciences, South China Agricultural University, No.483, Wushan Road, 510642 Guangzhou, China; 2 Guangdong Laboratory for Lingnan Modern Agricultural Science and Technology, South China Agricultural University, No.483, Wushan Road, Guangzhou, 510642 China; 3 College of Agronomy, Hunan Agricultural University, No.1, Nongda Road, Changsha, 410128 China

**Keywords:** Glycolate oxidase, H_2_O_2_, IAA, Photorespiration, Rice

## Abstract

**Background:**

Glycolate oxidase (GLO) is not only a key enzyme in photorespiration but also a major engine for H_2_O_2_ production in plants. Catalase (CAT)-dependent H_2_O_2_ decomposition has been previously reported to be involved in the regulation of IAA biosynthesis. However, it is still not known which mechanism contributed to the H_2_O_2_ production in IAA regulation.

**Results:**

In this study, we found that in *glo* mutants of rice, as H_2_O_2_ levels decreased IAA contents significantly increased, whereas high CO_2_ abolished the difference in H_2_O_2_ and IAA contents between *glo* mutants and WT. Further analyses showed that tryptophan (Trp, the precursor for IAA biosynthesis in the Trp-dependent biosynthetic pathway) also accumulated due to increased tryptophan synthetase β (TSB) activity. Moreover, expression of the genes involved in Trp-dependent IAA biosynthesis and IBA to IAA conversion were correspondingly up-regulated, further implicating that both pathways contribute to IAA biosynthesis as mediated by the GLO-dependent production of H_2_O_2_.

**Conclusion:**

We investigated the function of GLO in IAA signaling in different levels from transcription, enzyme activities to metabolic levels. The results suggest that GLO-dependent H_2_O_2_ signaling, essentially via photorespiration, confers regulation over IAA biosynthesis in rice plants.

**Supplementary Information:**

The online version contains supplementary material available at 10.1186/s12870-021-03112-4.

## Background

Photorespiration is the second-highest metabolic flux after photosynthesis in plants. It starts from the synthesis of 2-phosphoglycolate (2-PG) catalyzed by the oxygenase activity of ribulose-1,5-bisphosphate carboxylase/oxygenase (RuBisCO). 2-PG is immediately converted to glycolate in chloroplasts and transported to peroxisomes, where glycolate is detoxified into glycine by the glycolate/glyoxylate/glycine metabolic steps [1]. Glycolate oxidase (GLO, EC 1.1.3.15) is a key enzyme for the glycolate-glyoxylate conversion during photorespiration, which catalyzes the oxidation of glycolate to generate glyoxylate and H_2_O_2_ [2]. It has been estimated that the photorespiratory H_2_O_2_ produced by GLO may account for approximately 70% of the total H_2_O_2_ in C3 plants, thus making an important contribution to cellular redox status and participating in multiple H_2_O_2_-related processes [2]. GLO activity was reported to be up-regulated in pea, cowpea, and tobacco under drought stress [3–5]. More importantly, GLO-derived H_2_O_2_ can mediate nonhost resistance and gene-for-gene-mediated resistance in *Arabidopsis thaliana* and *Nicotiana benthamiana* [6]*,* and it is also involved in *barley stripe mosaic virus* infection in barley and basal defense against *Pseudomonas syringae* in tomato [7, 8]. In addition, initiation of the systemic acquired acclimation and formation of iron plaques on the surface of roots in plants is mediated by GLO-derived H_2_O_2_ [9, 10].

H_2_O_2_, as a typical reactive oxygen species, is not only biologically toxic but also serves as an important signaling molecule. Its homeostasis is elaborately regulated by the balance between the cellular generation and scavenging rates [11]. Photorespiratory H_2_O_2_ is mainly scavenged by peroxisomal catalase (CAT, EC 1.11.1.6), and GLO and CAT usually act in concert to regulate intracellular photorespiratory H_2_O_2_ levels in plants [11]. It has been observed that CAT-dependent H_2_O_2_ cross-talks with other phytohormone signaling pathways. For instance, ABA regulates the expression of peroxisomal CAT and hence the levels of H_2_O_2_ in water-stressed *Arabidopsis* [12]. Another potential point of crosstalk is between the CAT-dependent H_2_O_2_ and IAA signaling pathway, which has recently gained much attention. Gao et al. [13] found that the absence of the photorespiratory CAT2 resulted in accumulation of the photorespiratory H_2_O_2_. Additionally, increased H_2_O_2_ suppressed the IAA synthesis in *Arabidopsis*. In turn, exogenous auxin activated IAA signaling to counteract photorespiratory H_2_O_2_-dependent cell death in *Arabidopsis cat2* mutants [14]. Yuan et al. [15] further demonstrated that the accumulated H_2_O_2_ in *Arabidopsis* resulting from CAT2 inhibition promoted the sulfenic acid modification of tryptophan synthetase β (TSB) subunit 1, subsequently decreasing TSB activity to repress the synthesis of the IAA precursor Trp. While CAT-dependent H_2_O_2_ decomposition intertwined with the IAA signaling pathway, the molecular details of this process remain scarce, and more notably, it is not clear whether GLO dependent H_2_O_2_ production plays a major role in IAA regulation.

As an important engine for H_2_O_2_ production, GLO has various isoforms that are encoded by multi-gene families in plants. *AtGOX1* and *AtGOX2* are the major isoforms involved in basic photorespiration metabolism in *Arabidopsis thaliana* [6]. We have earlier demonstrated that *OsGLO1* and *OsGLO4* are the dominant isoforms for photorespiration in rice [16, 17]. In this study, in order to understand whether and how GLO-dependent H_2_O_2_ modulates IAA signaling, various genetically-modified rice lines of *GLO1* and *GLO4* were generated. It was found that IAA levels in *glo1* and *glo4* knockout mutants were significantly up-regulated which was correlated with lower peroxisomal H_2_O_2_ levels. Further analyses showed that IAA and Trp contents were increased, and that TSB activity and the genes related to Trp-dependent IAA biosynthesis and IBA to IAA conversion were also up-regulated. The results suggest that GLO-dependent H_2_O_2_ production can regulate IAA biosynthesis in rice.

## Results

### Rice GLO mutants exhibited photorespiration phenotypes as accompanied with decreased peroxisomal H_2_O_2_

**Fig. 1:**
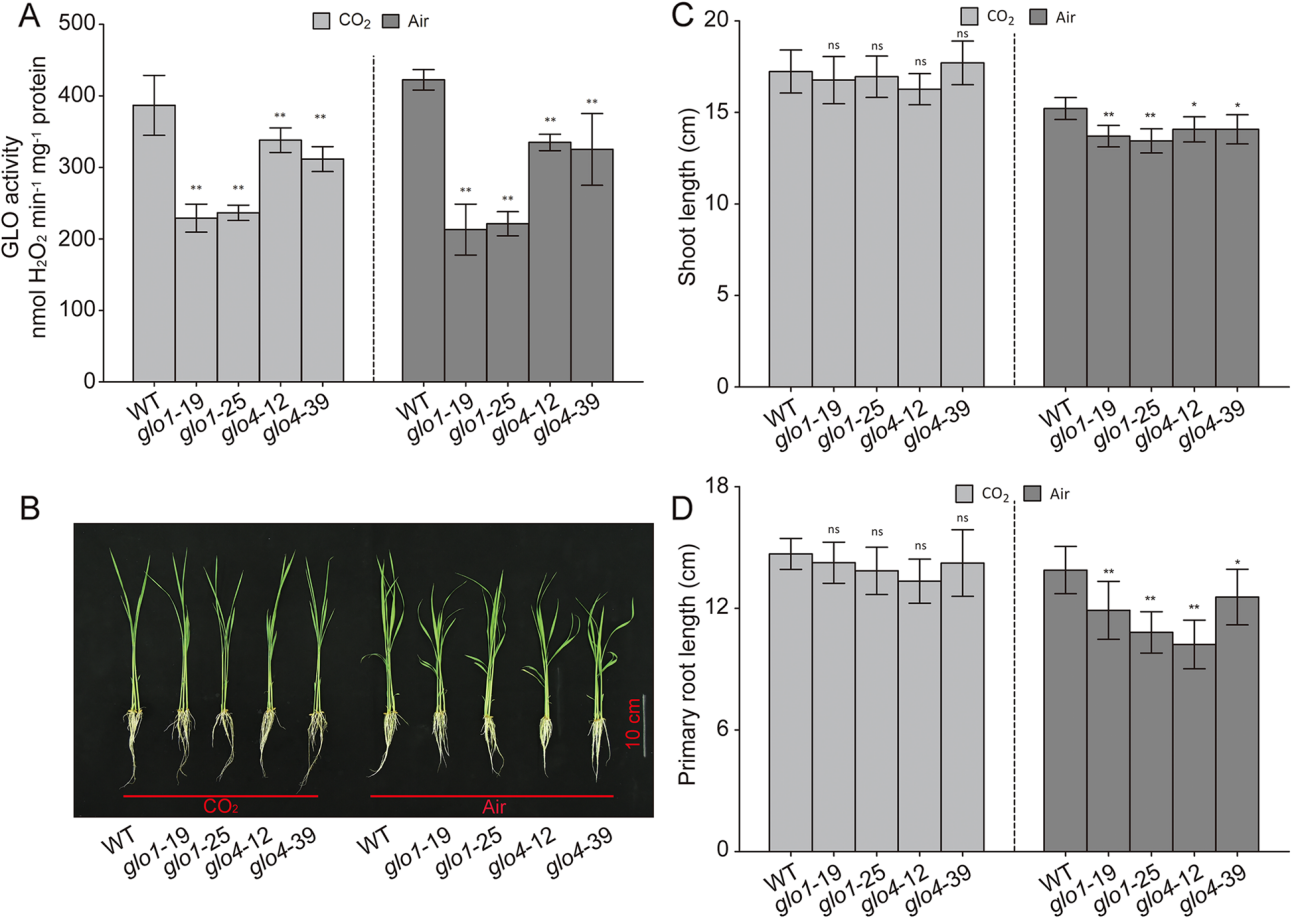
GLO activity and growth phenotype of *glo1* and *glo4* mutants. Germinated seeds of *glo1* and *glo4* mutants were divided into two groups and cultured in two growth chambers under atmospheric and high CO_2_ (3500 ppm) conditions. The seedlings were then used for GLO activity measurement (**A**), phenotypic photo (**B**), shoot length and primary root length statistics (**C**, **D**). Results are representative of three independent experiments. Data are presented as means ± SD of three biological replications, *P < 0.05, **P < 0.01 according to Student’s *t*-tests

**Fig. 2:**
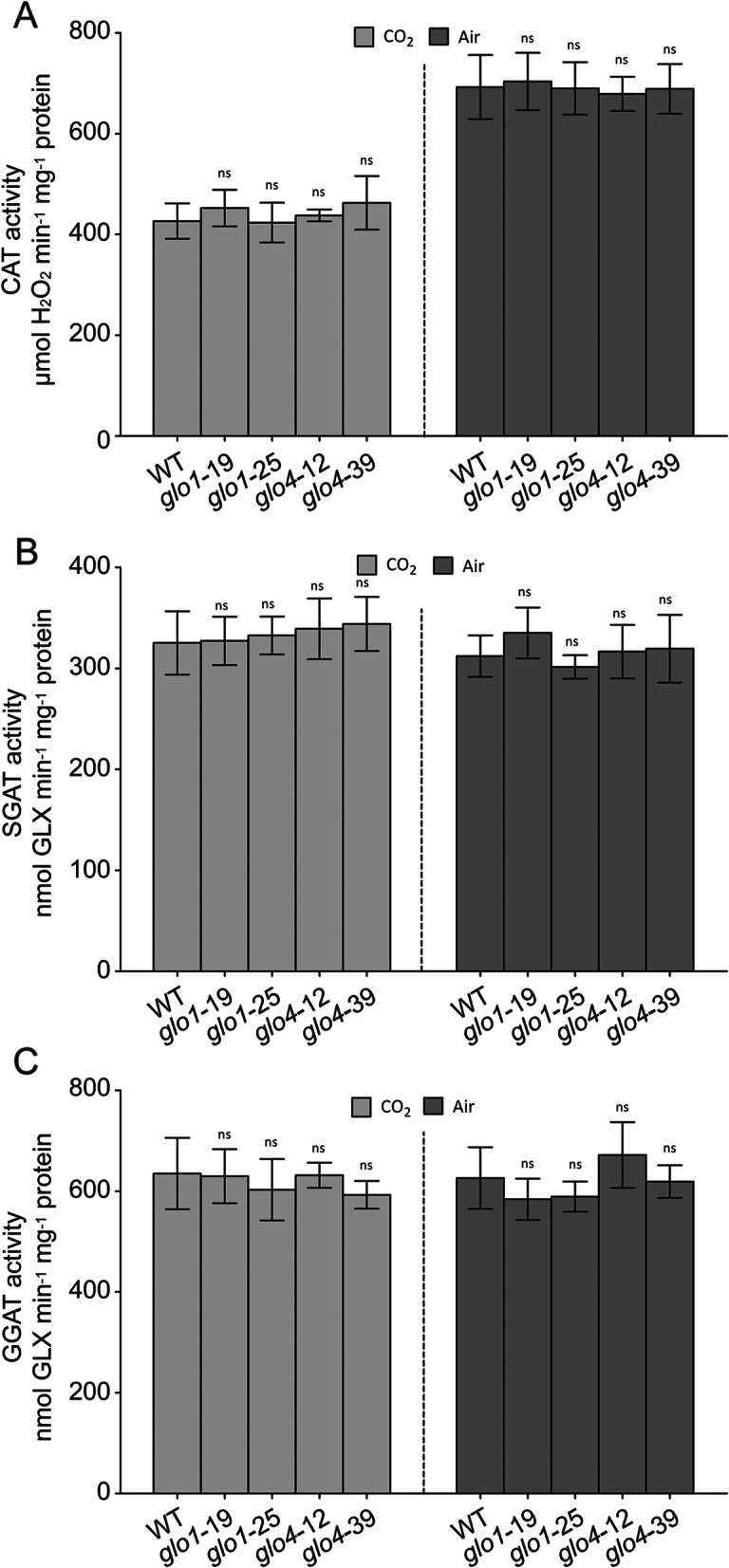
The activity of CAT, SGAT and GGAT in *glo1* and *glo4* mutants. Germinated seeds of *glo1* and *glo4* mutants were cultured under atmospheric and high CO_2_ (3500 ppm) conditions. The seedlings were then used for CAT (**A**), SGAT (**B**) and GGAT (**C**) activity measurement. Data are presented as means ± SD of three biological replications, *p < 0.05, **p < 0.01 according to Student’s *t*-test

The rice genome contains four *GLO* homologs, i.e., *GLO1* (Os03g0786100), *GLO3* (Os04g0623500), *GLO4* (Os07g0152900), and *GLO5* (Os07g0616500). *GLO1* and *GLO4* are the primary *GLO* genes that contribute to photorespiratory glycolate-glyoxylate metabolism [16, 17]. In this study, knockout and overexpression lines of *GLO1* and *GLO4* were generated in rice. Western blot analysis confirmed that GLO1 and GLO4 were absent in the *glo1* and *glo4* knockout mutants, respectively (Additional file 1A, B). GLO activities were decreased by approximately 55% and 30% in the *glo1* and *glo4* mutants, respectively. In *GLO* overexpression (*GLO*OE) lines, GLO activities were increased by 60–95% in *GLO1*OE lines and 40–55% in *GLO4*OE lines (Fig. 1A and Additional file 2A). Both seedlings of *glo1* and *glo4* knockout mutants showed dwarfish growth as compared with WT but recovered to normal stature when photorespiration was suppressed under high CO_2_ condition (Fig. 1B, C and D). The growth phenotypes of those overexpression lines have been described earlier in our previous study. Briefly, the growth of *GLO*OE lines was improved when GLO activities were increased by 60–100%, whereas reduced growth was noticed when GLO activity was increased over 150% [18]. In addition, some downstream enzymes of GLO, such as SGAT and GGAT, were little altered in all GLO modified lines under both ambient and high CO_2_ conditions (Fig. 2 and Additional file 2). The CAT activity was suppressed by 30 to 40% under high CO_2_, but there was no difference in CAT activities between various GLO modified lines under ambient and high CO_2_ conditions (Fig. 2A and Additional file 2B). This is consistent with previous studies showing that GLO downstream steps in photorespiration are not significantly affected by GLO modification [19–21].

**Fig. 3:**
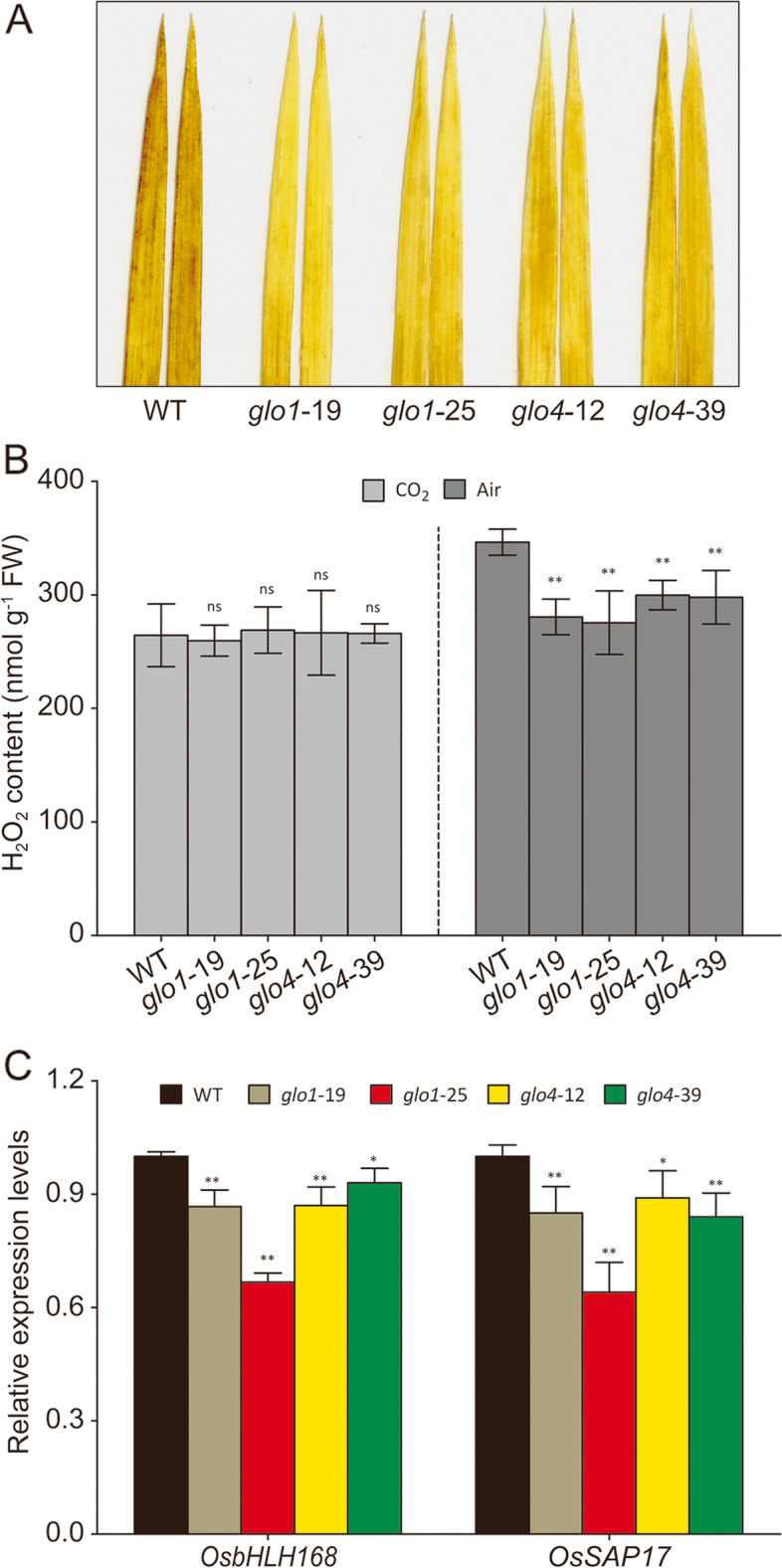
Detection of H_2_O_2_ levels in *glo1* and *glo4* mutants. H_2_O_2_-DAB staining of rice leaves under atmospheric condition (**A**); endogenous H_2_O_2_ contents of rice leaves under atmospheric and high CO_2_ condition (**B**); qRT-PCR analysis of peroxisomal H_2_O_2_-responsive genes in *glo1* and *glo4* mutants under atmospheric condition (**C**), the two H_2_O_2_ indicators selected were previously identified peroxisome-specific H_2_O_2_-responsive genes, *OsbHLH168* (Os01g0108600) and *OsSAP17* (Os09g0385700). Results are representative of three independent experiments. Data are presented as means ± SD of three biological replications, *P < 0.05, **P < 0.01 according to Student’s *t*-tests

As the fastest pathway for H_2_O_2_ production, photorespiratory H_2_O_2_ is mainly generated by the GLO-catalyzed glycolate oxidation reaction [1, 2]. The absence of *GLO1* and *GLO4* can significantly decrease H_2_O_2_ levels in rice leaves under atmospheric condition (Fig. 3A, B), and this difference disappeared under high CO_2_ (Fig. 3B). No significant changes were observed in *GLO1* and *GLO4* overexpression lines under both atmospheric and high CO_2_ conditions (Additional file 3A, B). To further prove that the decreased H_2_O_2_ levels occur in peroxisomes, we quantified the transcript abundance of two previously identified peroxisome-specific H_2_O_2_-responsive genes, *OsbHLH168* (Os01g0108600) and *OsSAP17* (Os09g0385700) [22, 23], by qRT-PCR. As shown in Fig. 3C, transcript levels of both *OsbHLH168* and *OsSAP17* were significantly lowered in the *glo1* and *glo4* mutants under atmospheric condition. For *GLO* overexpression lines, the expression levels of these two genes were not significantly different (Additional file 3C). These results indicated that the decreased H_2_O_2_ levels occurred in peroxisomes, due to reduced GLO activities in *glo1* and *glo4* mutants.

### The GLO-dependent H_2_O_2_ production regulated IAA and Trp levels

**Fig. 4:**
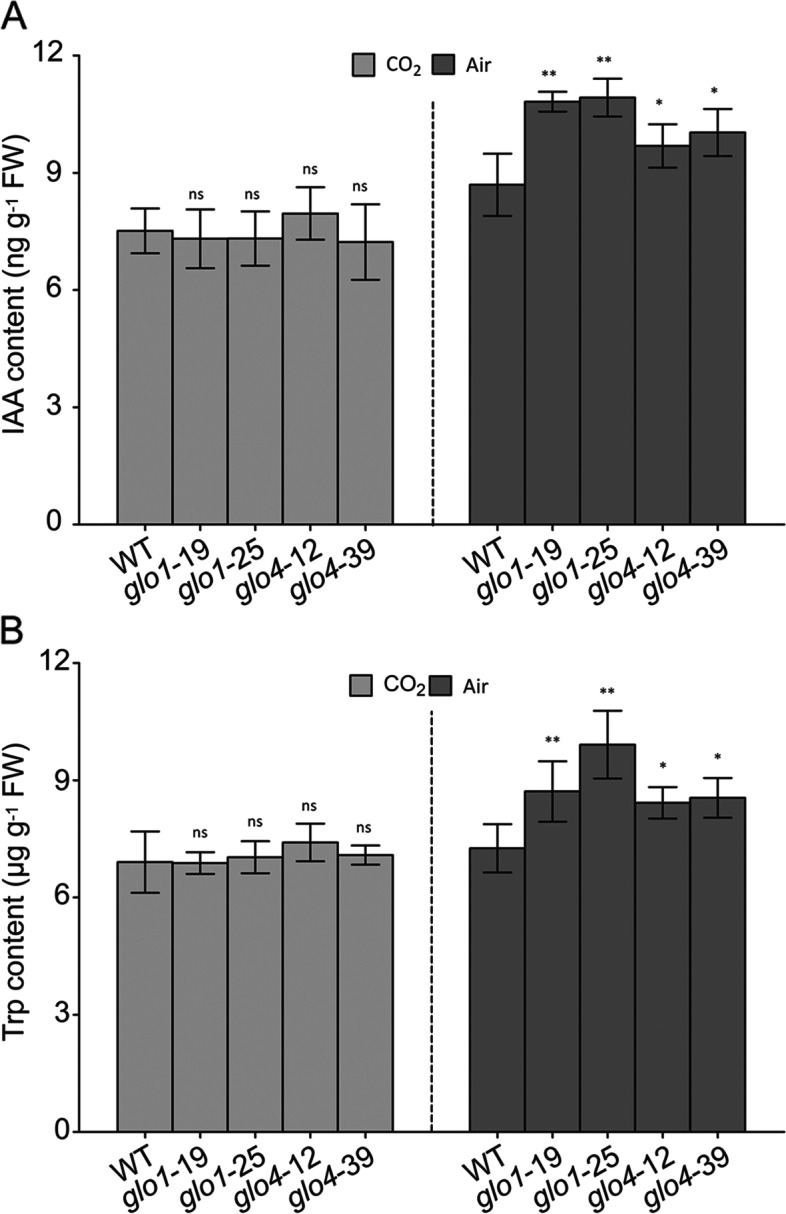
Changes of IAA and Trp levels in *glo1* and *glo4* mutants. Germinated seeds were cultured under atmospheric and high CO_2_ conditions. The leaves of five-leaf stage rice seedlings were then detached for determining IAA (**A**) and Trp (**B**). Data are presented as means ± SD of three biological replications, *P < 0.05, **P < 0.01 according to Student’s *t*-tests

It was previously reported that the CAT-dependent H_2_O_2_ accumulation can suppress IAA synthesis in *Arabidopsis* [13, 15]. Since CAT and GLO may work cooperatively [11], it is of interest to further understand whether GLO is involved in the H_2_O_2_-IAA crosstalk. As the peroxisomal H_2_O_2_ levels were decreased (Fig. 3), the IAA contents were increased by 25–30% and 15–20% in the *glo1* and *glo4* knockout mutants, respectively (Fig. 4A). No obvious changes in IAA were observed in overexpression lines (Additional file 4A), in which H_2_O_2_ levels were unchanged (Additional file 3). Trp is the primary precursor for IAA biosynthesis in plants [24, 25], so we further determined if Trp contents were affected in various *GLO* genetically-modified rice lines. As shown in Fig. 4B, Trp content was about 6.5 µg g^−1^ FW in WT, which was increased to 8.5–10.0 µg g^−1^ FW in *glo1* knockout mutants, and 8.0–8.5 µg g^−1^ FW in *glo4* knockout mutants. No obvious changes were detected in the overexpression lines (Additional file 4B). Also, Trp contents were changed with a similar tendency as IAA. When GLO-dependent H_2_O_2_ production was suppressed under high CO_2_ condition, the differences in IAA and Trp contents in *glo1* and *glo4* knockout mutants vanished (Fig. 4A, B). The above results indicated that the reduced photorespiratory H_2_O_2_ resulting from the reduction of GLO activity was correlated with increased IAA and Trp contents in rice.

### The regulation of IAA occurred at the biosynthetic level

**Fig. 5:**
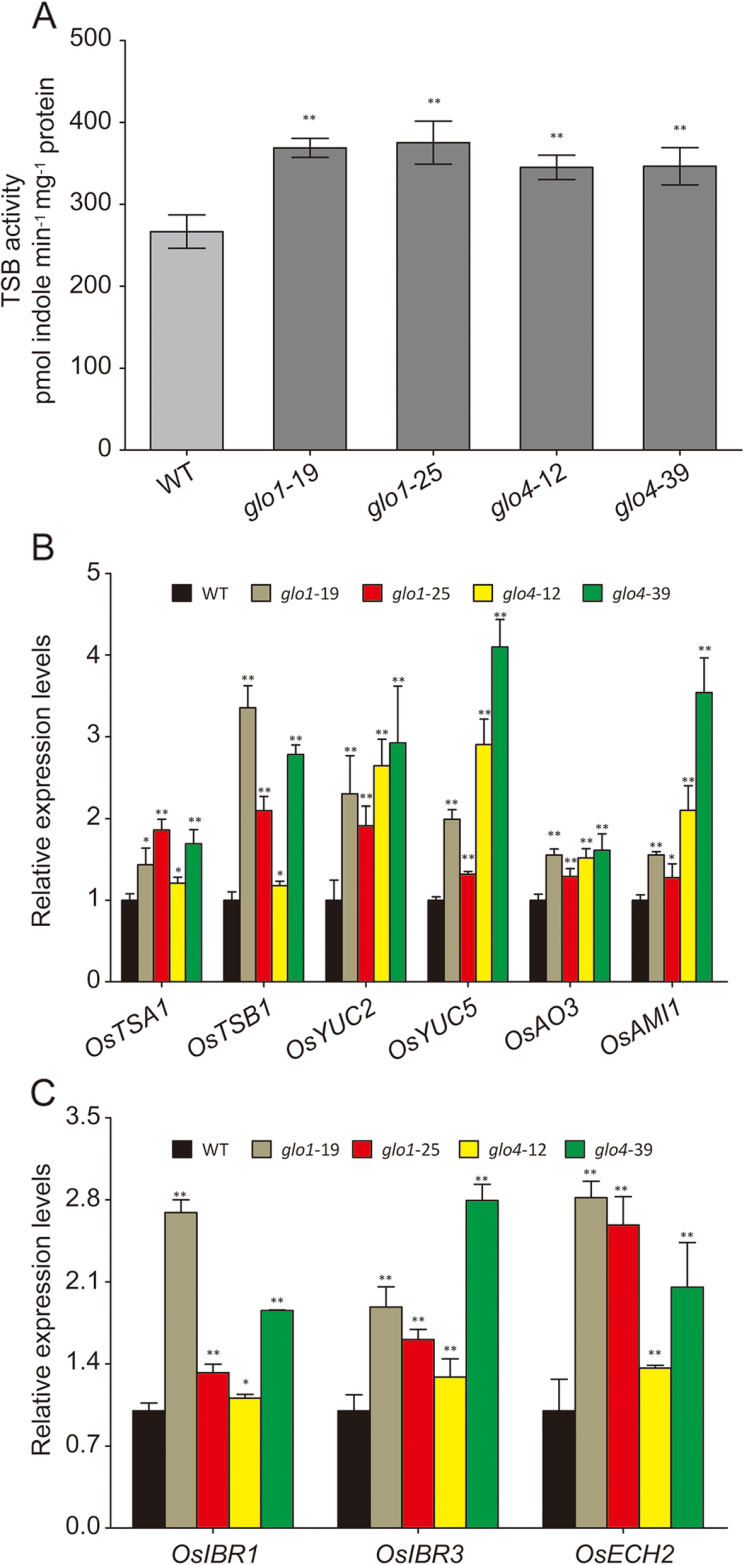
TSB activity and expression patterns of IAA synthesis-related genes in *glo1 and glo4* mutants. (**A**) TSB activity in *glo* mutants. **(B)** Expression patterns of genes participating in Trp biosynthesis and Trp-dependent IAA biosynthesis**.** (**C**) Expression patterns of genes involved in the conversion of IBA to IAA. The leaves of five-leaf stage rice seedlings grown in atmospheric condition were detached for TSB activity measurement and RNA extraction. Data are presented as means ± SD of three biological replications, *P < 0.05, **P < 0.01 according to Student’s *t*-tests

Theoretically, IAA content may be regulated at the levels of biosynthesis and sequestration [26]. The Trp-dependent pathway has been known to dominate IAA biosynthesis, so we first detected TSB activities that are responsible for the biosynthesis of the IAA precursor Trp. As shown in Fig. 5A, TSB activities were increased by 40% and 27% in *glo1* and *glo4* mutants, respectively. No changes were observed in *GLO* overexpression lines (Additional file 5). Correspondingly, transcripts of the two subunits of tryptophan synthase (β subunit 1, *TSB1*; α subunit 1, *TSA1*) were also significantly enhanced in *glo* mutants (Fig. 5B). Besides, some other downstream genes involved in the Trp-dependent IAA biosynthesis (Additional file 6), i.e. *OsYUC2* (Os01g0732700), *OsYUC5* (Os07g0437000), *OsAO3* (Os03g0790700), and *OsAMI1* (Os04g0118100), were still up-regulated in *glo* mutants (Fig. 5B).

The conversion of IBA to IAA occurr in the peroxisome, which could be intimately regulated by the redox state of peroxisomes [27, 28]. Hence, the expressions levels of those genes proposed to be involved in the conversion of IBA to IAA were determined (Additional file 6), i.e. *OsIBR3* (Os07g0675133), *OsECH2* (Os09g0544900), and *OsIBR1* (Os09g0133200). These data showed that all of these genes were up-regulated in *glo* mutants (Fig. 5C).

**Fig. 6:**
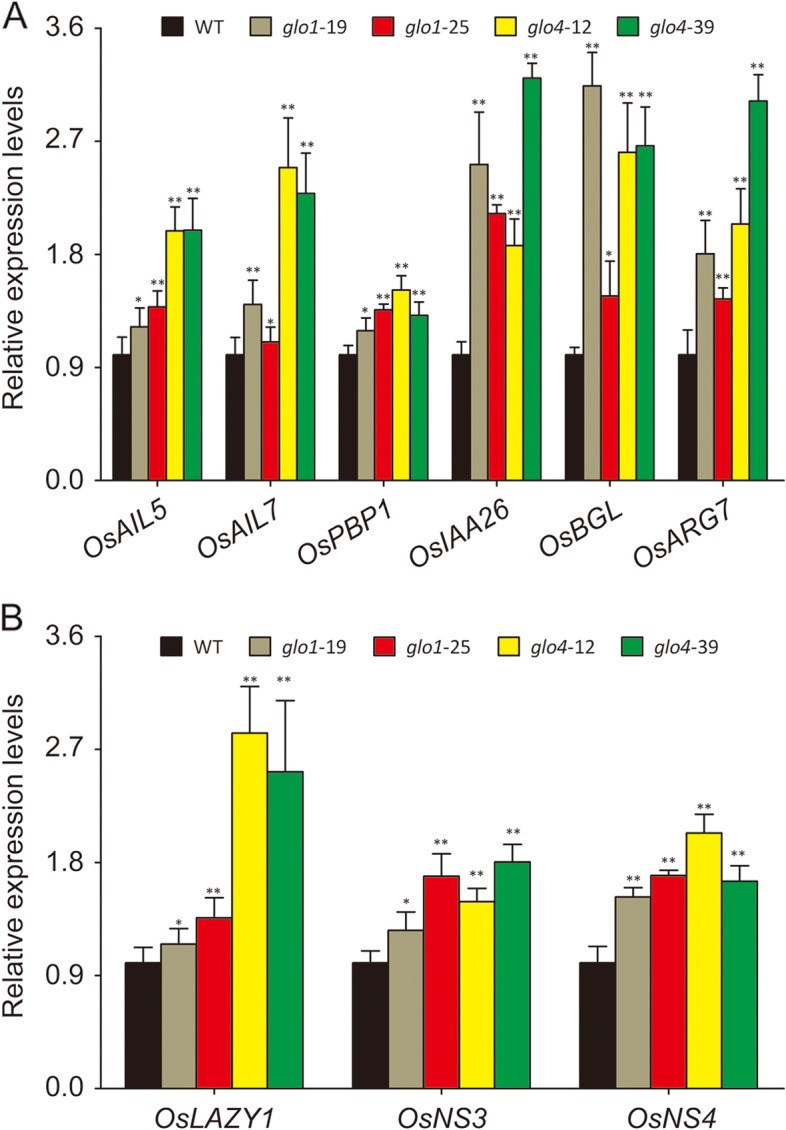
Expression patterns of IAA responsive/transport genes in *glo1* and *glo4* mutants. (**A**) Expression patterns of genes in response to IAA content. (**B**) Expression patterns of genes involved in IAA transport. The leaves of five-leaf stage rice seedlings grown in atmospheric condition were detached for RNA extraction. Data are means ± SD of three biological replications, *P < 0.05, **P < 0.01 according to Student’s *t*-tests

Finally, the expression levels of various IAA-responsive/transport genes, which are known to be regulated in an IAA-dependent manner, were analyzed (Additional file 7). As expected, the genes involved in IAA responsive and transport processes, i.e. *OsAIL5* (Os04g0653600), *OsAIL7* (Os03g0313100), *OsPBP1* (Os01g0783700), *OsIAA26* (Os09g0527700), and *OsLAZY1* (Os11g0490600), *OsNS3* (Os10g0147400), *OsNS4* (Os11g0169200) were transcriptionally up-regulated in *glo* mutants (Fig. 6A, B), and these genes may further function in multiple plant growth processes and stress responses. The above results collectively indicate that the GLO-dependent H_2_O_2_ production regulates IAA biosynthesis to perform various biological functions in rice.

## Discussion

In recent decades, information has become increasingly available indicating that photorespiration can influence various biological processes, e.g., carbon metabolism, energetics, nitrogen assimilation, and respiration [29–31]. Remarkably, photorespiration is a major source for H_2_O_2_ in photoautotrophic tissues, thereby making important contributions to cellular redox status and signaling [2, 11, 30]. Reports indicate that H_2_O_2_ is closely associated with IAA during plant development, and that IAA can facilitate H_2_O_2_ accumulation in the roots of maize, tomato, and *Arabidopsis* to regulate root growth and gravitropism [32–34], while IAA biosynthesis and signaling were inhibited by both exogenous and endogenous H_2_O_2_ in *Arabidopsis* seedlings [35, 36]. Nevertheless, how H_2_O_2_ production and signaling interact with IAA is still not well understood. Gao et al. [13] demonstrated that the CAT-dependent H_2_O_2_ was involved in the regulation of IAA, but no direct evidence was provided as to whether the H_2_O_2_ is derived from photorespiration, because CAT-dependent H_2_O_2_ may come from other sources in the peroxisome, such as fatty acid β-oxidation and dismutation of O_2_^.−^ [37]. By using *GLO* knockout mutants, we further demonstrated that the GLO-dependent H_2_O_2_ also regulated IAA levels in rice (Fig. 3 and Fig. 4A). However, overexpression of either *GLO1* or *GLO4* conferred no significant changes in H_2_O_2_ levels (Additional file 3), or on IAA contents (Additional file 4A). It has been similarly noticed that overexpression of GLO conferred no enhanced effects on photorespiratory flux and H_2_O_2_ production, considering that several photorespiratory enzymes (e.g. GLO, 2-phosphoglycolate phosphatase) are sufficiently high to manage the metabolic flux of photorespiration under normal conditions [18, 38] and further up-regulation is only beneficial under some stress environments [3–5, 8]. Overall, our present results suggest that GLO-dependent H_2_O_2_ regulates IAA levels in rice, not only supporting the results from the *Arabidopsis cat2* mutants [13, 14], but also providing direct evidence that the CAT-dependent H_2_O_2_ ultimately comes from the GLO-catalyzed reaction, essentially via photorespiratory metabolism in plants.

Two IAA biosynthetic strategies exist in plants, namely, Trp-independent and Trp-dependent pathways. Indole-3-glycerol phosphate and indole are the likely precursors of Trp-independent IAA biosynthesis, but its complete biochemical pathway has not yet been elucidated. Trp is the precursor in the Trp-dependent IAA biosynthesis, and several pathways have been proposed for the IAA biosynthetic strategy, but molecular and genetic evidence of the key enzymes involved in such pathways are still not confirmed [24, 25]. Tryptophan synthetase β (TSB), which catalyzes Trp formation from Ser and indole, is a key limiting factor in Trp biosynthesis. It has also been documented that elevated H_2_O_2_ content in *Arabidopsis* leaves decreased TSB activity, thus reducing Trp and IAA levels [15, 39]. Our present study showed that the increased TSB activity (Fig. 5A) and up-regulated expression of genes responsible for the Trp-dependent IAA biosynthesis (Fig. 5B) were positively correlated with the increased levels of Trp and IAA in *glo* mutants (Fig. 4A, B). These lines of evidence suggest that the Trp-dependent pathway may have dominated in the IAA biosynthesis of rice plants that is regulated by GLO-dependent H_2_O_2_. In addition, modifications in IAA can alter its activity and sequester active IAA [40]. For example, IBA is a chain-elongated form of IAA and functions as a pool for sequestering IAA, which may still be an efficient mechanism for IAA regulation in plants once IBA is converted to IAA [27, 40, 41]. Altering the pool of IAA derived from IBA is known to result in a set of developmental defects in *Arabidopsis* [42, 43]. IBA is likely converted to IAA through a β-oxidation pathway in the peroxisome [27, 40], where H_2_O_2_ might serve as a key regulator [28]. Although we failed to accurately determine IBA contents due to technical difficulties [27, 44], the expression levels of the genes involved in the IBA to IAA conversion were shown to be significantly up-regulated in the *glo* mutants (Fig. 5C). Therefore, it is likely that the elevated IAA levels caused by decreased H_2_O_2_ levels in *glo* mutants may partially be contributed by the increased conversion of IBA to IAA.

**Fig. 7:**
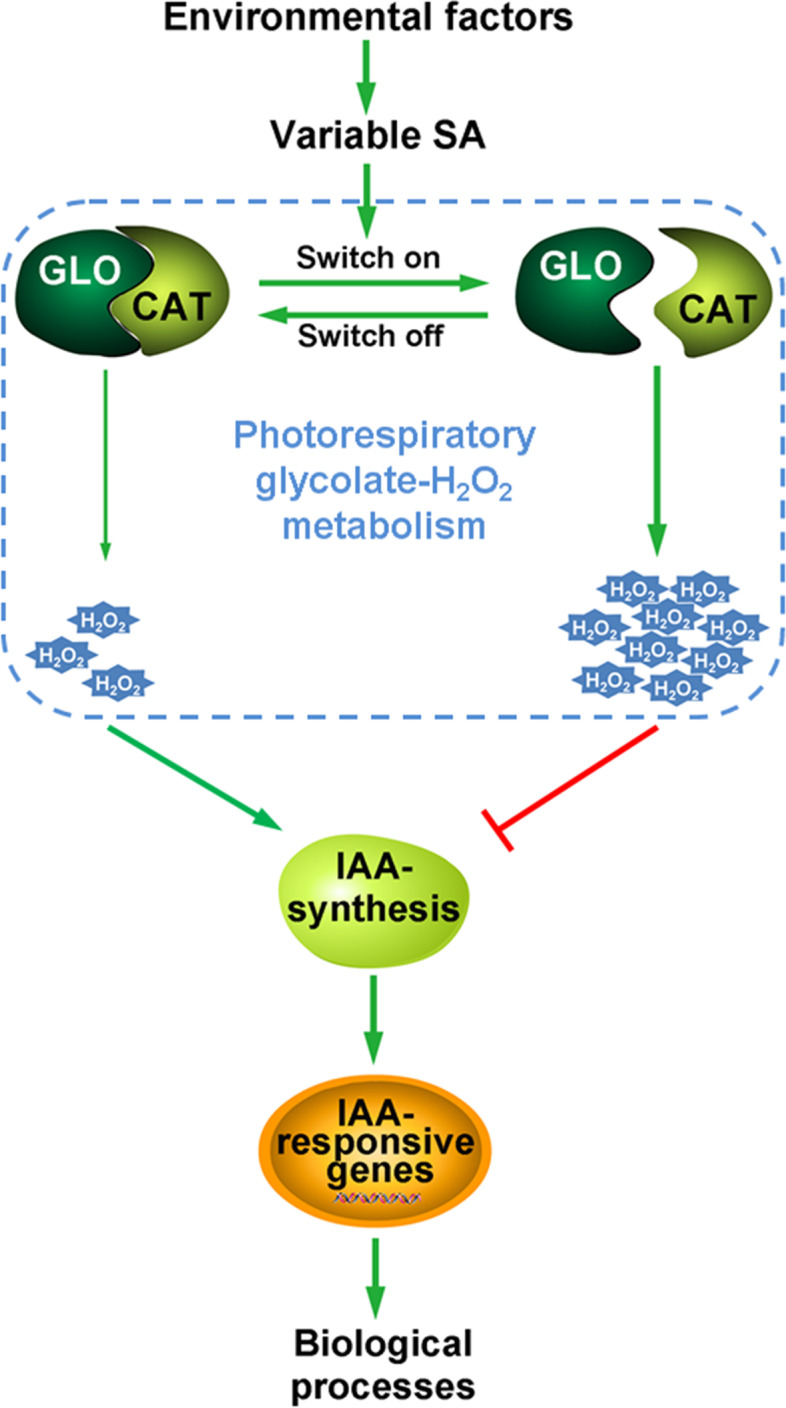
A proposed model of how SA links to IAA via photorespiration. This model was formed by referring to Zhang et al., [11]. When GLO is interacted with CAT, the photorespiratory H_2_O_2_ produced by GLO could be quickly removed by CAT through substrate channeling mechanism, otherwise the photorespiratory H_2_O_2_ will be accumulated. The dynamic interaction of GLO and CAT acts as a molecular switch to sense variable SA levels in response to environmental changes. The photorespiratory H_2_O_2_ fluctuation generated by the association-dissociation of GLO and CAT could further regulate IAA biosynthesis

It is well known that IAA signaling controls numerous plant growth and development processes by regulating the expression of various IAA response factors/genes. Indeed, the expression of many IAA-responsive/transport genes (Additional file 7) was markedly up-regulated in *glo1* and *glo4* mutants (Fig. 6A, B). Our current data, in combination with the results from other studies [13, 15], strongly support that IAA levels can be regulated by either CAT or GLO in plants. IAA is a key phytohormone responding to various environmental stresses, and growth inhibition by decreased IAA levels is an important physiological strategy for plants in response to drought and pathogen infection [45, 46]. Meanwhile, GLO can be induced by drought, high temperature and pathogen infection [3, 5, 8], and these stresses can also result in salicylic acid (SA) accumulation in plants [47–49]. We have previously proposed a switch model, in which a physical association-dissociation of GLO and CAT, in response to stimuli such as SA, serves as a mechanism to modulate cellular H_2_O_2_ levels [11]. More interestingly, it has been documented that SA could negatively regulate the synthesis and/or signaling of IAA, they act in a mutually antagonistic manner in plants [50–53], and a recent study further suggested that SA regulation of IAA is mediated by H_2_O_2_ [15]. Therefore, we speculate that SA may modulate H_2_O_2_ through our proposed switch model, subsequently conferring regulation on IAA. Moreover, the regulation of H_2_O_2_ may occur in a fluctuating manner because the association-dissociation of GLO and CAT could take place dynamically and transiently in response to environmental stresses or stimuli, e.g., drought and pathogen infection. Therefore, we here further modified our proposed model, in which the switch-modulated H_2_O_2_ fluctuation confers biological functions via regulating IAA levels in plants (Fig. 7).

## Conclusion

In this study, we found that decreases in GLO-dependent H_2_O_2_ levels were accompanied with IAA and Trp accumulation in *glo* rice mutants, whereas high CO_2_ was able to abolish the IAA difference. Subsequent analyses of transcript levels and enzyme activities of genes involved in IAA metabolism suggest that both Trp-dependent IAA biosynthesis and IBA to IAA conversion contribute to the increase of IAA contents. This data offer insights into how GLO-dependent H_2_O_2_, essentially via photorespiration, regulates IAA biosynthesis in plants. Furthermore, taken together with our previously reported association-dissociation mechanism of GLO and CAT, we further consider that H_2_O_2_ waves as regulated by SA via such a proposed mechanism may be an important point for crosstalk between SA and IAA. However, molecular details of this signalling pathway and its biological significance remain an important issue for our future research.

## Methods

### Plant materials, growth conditions, and treatments

*Oryza sativa* cv. Zhonghua 11 (japonica cultivar-group) preserved by our laboratory was used for the functional analyses and *GLO* transgenic line construction [20]. Pre-germinated rice seeds were grown in Kimura B complete nutrient solution under natural conditions. After reaching the four-leaf stage, seedlings were transplanted, either being continuously grown in Kimura B complete nutrient solution in a plant growth chamber with a light cycle of 14 h light/10 h dark (30 °C / 25 °C, respectively) at 600 µmol photons m^−2^ s^−1^ on average, relative humidity 60%-80%, or grown in paddy fields under natural conditions. For high concentration CO_2_ treatment, pre-germinated rice seeds were cultured in Kimura B complete nutrient solution in a plant growth chamber (Percival E-41HO, USA) supplied with 3500 ppm CO_2_ [30, 54, 55].

### Generation of genetically modified rice lines

Total RNA was extracted using the Plant RNA Purification Reagent (Magen Biotech), cDNA was prepared from 1 μg of total RNA using HiScript® II Reverse Transcriptase (Vazyme) as recommended. To generate *GLO* overexpression transgenic lines, each *GLO* sequence was cloned into the pYLox.5 vector using the ClonExpress Ultra One Step Cloning Kit (Vazyme) [56]. For the generation of CRISPR-Cas9 knockout lines, specific targeting sequences of *GLO1* and *GLO4* (Additional file 8) were synthesized and cloned into the pYLCRISPR/Cas9Pubi vector [57]. The constructed vectors were transformed into rice callus using *Agrobacterium*-mediated infection (strain *EHA105*). The T1 seeds from the positive T0 lines were germinated and then transplanted into soil to grow until the T2 seeds were harvested. After that, the T2 homozygous plants that originated from two independent lines were used for subsequent treatments and analyses.

### Enzyme assays and protein measurement

#### GLO activity assay

GLO activity was detected in an enzyme coupled assay [16]. Briefly, 0.1 g leaves were detached from the youngest fully expanded leaves (five-leaf stage) and homogenized in 1 mL of 50 mM PBS (pH 7.4) at 4 °C, the homogenates were centrifuged at 12 000 g for 20 min at 4 °C and the supernatants were used as enzyme extracts. The 1 mL reaction mixture containing 50 mM PBS (pH 7.8), 1 mM 4-amino-antipyrine, 0.1 mM FMN, 2 mM phenol, 5 units of horseradish peroxidase, and 5 mM glycolate. The reaction was started by adding enzyme and the absorbance at 520 nm was recorded at intervals of 5 s for 1 min. The protein content was determined using Coomassie brilliant blue G250.

#### TSB activity assay

TSB activity was measured according to Last et al. [39]. All steps were performed at 4 °C, unless indicated otherwise. Plant extracts were prepared by grinding 1 g of leaves from five-leaf stage rice seedlings into a paste with a prechilled mortar and pestle containing 1.5 mL of 0.1 M K-phosphate (pH 8.2), 0.3 g quartz sand, and 0.1 g polyvinylpolypyrrolidone. Homogenates were then centrifuged twice at 12,000 g for 15 min and the supernatants were used as the enzyme extracts. The 1 mL reaction solution was prepared containing 60 mM L-serine, 0.2 mM indole, 80 mM potassium phosphate (pH8.2), 10 μg pyridoxal phosphate, and 0.4 mL plant extract with gentle agitation at 30 °C. The reaction was stopped by the addition of 0.1 mL of 0.2 M sodium hydroxide after 90 min. The residual indole was extracted into 4 mL of toluene by gentle vortexing (vigorous agitation created a permanent emulsion). After centrifugation for 15 min at 1500 g, 0.5 mL of the toluene layer was added to 2 mL ethanol and 1 mL Ehrlich’s Reagent (Sigma). The color could develop for 30 min at room temperature and the absorbance of the product was measured spectrophotometrically at 540 nm.

#### SGAT and GGAT activities assay

SGAT and GGAT activities were measured by detecting the reduction of glyoxylate according to Yu et al. [58] with some modifications. Briefly, 50 mg of leaves (five-leaf stage) were homogenized in 1 mL 50 mM K-phosphate (pH 7.4) at 4℃, and the homogenate was then centrifuged at 12 000 g and 4℃ for 15 min. The supernatant was used as enzyme extract. The 0.5 mL enzymatic reaction mixture containing 50 mM K-phosphate (pH7.4), 50 mM glyoxylate, 5.5 mM pyridoxal-5-phosphate and appropriate enzyme extract. The enzymatic reaction was started by adding 20 mM L-serine for SGAT or 20 mM L-glutamate for GGAT at 30℃ for 20 min. This enzymatic reaction was terminated by adding 0.1 mL 2 M HCl and neutralized with 0.1 mL 2 M NaOH. After that, 0.1 mL 0.33% phenylhydrazine hydrochloride was added to the mixture and incubated at 30℃ for 15 min. Finally, 0.5 mL HCl and 0.1 mL 1.65% potassium ferricyanide was added for color reaction, the red color produced was measured at 520 nm.

#### CAT activity assay

The CAT activity was detected using a UV spectrophotometer in a reaction mixture containing 50 mM PBS (pH 7.4), 25 mM H_2_O_2_ at 30℃. The consumption of H_2_O_2_ was measured at 240 nm and the CAT activity was calculated using the extinction coefficient for H_2_O_2_ of 43.6 M^−1^ cm^−1^ [11].

### 3, 3′-diaminobenzidine (DAB) staining for H_2_O_2_ detection

The leaf H_2_O_2_ abundance was determined in situ by DAB uptake methods [11]. The youngest fully expanded leaves (10 cm, at the five-leaf stage) were detached, and the cut end was dipped into DAB solution (1 mg ml^−1^, pH 3.8) for 2 h in a growth chamber (light intensity 700 μmol m^−2^ s^−1^, temperature 25 °C, and relative humidity 60%). After that, the leaves were de-stained twice with ethanol and photographed.

### Assay of H_2_O_2_ content

Endogenous H_2_O_2_ content in rice leaves (at five-leaf stage) were measured using an Amplex Red H_2_O_2_/peroxidase assay kit (Invitrogen, USA) [11]. Briefly, 0.1 g leaves were detached and immediately ground in liquid nitrogen, and then the powder was extracted in 1 ml PBS (50 mM, pH 7.4) and centrifuged at 12 000 g for 15 min at 4℃. The supernatant was used to determine H_2_O_2_ levels.

### Quantification of IAA

For IAA quantification, 1 g of leave material was detached from the youngest fully expanded leaves (five-leaf stage) and immediately frozen in liquid nitrogen. The extraction and quantification of endogenous IAA were conducted according to the manufacturer’s instructions (Wuhan Metware Biotechnology Co., Ltd., Wuhan, China) [59]. Briefly, 50 mg of frozen leaf material was ground into a powder and extracted with methanol/water/formic acid (15:4:1, V/V/V). The combined extracts were evaporated to dryness under a stream of nitrogen gas, were reconstituted in 80% methanol (V/V), and filtered (PTFE, 0.22 μm, Anpel) before LC–MS/MS analysis. The quantification of IAA was conducted using an ultra-performance liquid chromatography-tandem mass spectrometry (LC–MS/MS) system (UPLC, Shim-pack UFLC SHIMADZU CBM30A system, Kyoto, Japan; MS, Applied Biosystems, Foster City, CA). The content of IAA was determined using an external standard method and was expressed as ng/g fresh weight (FW). Three biological replications were performed per sample.

### Determination of Trp content

Measurement of Trp content was based on a method reported previously with some modifications [60]. The youngest fully expanded leaves were detached and frozen in liquid nitrogen immediately for subsequent analysis. Harvested leaves were sampled at 0.5 g each and homogenized in 3 mL of 2% (w/v) sulphosalicylic acid. After incubating at 25 °C for 2 h, the homogenates were centrifuged at 12 000 g for 20 min, and then the supernatants were filtered through a 0.22 μm nylon membrane. Trp contents in the filtrates were determined with a high-speed automatic amino acid analyzer (Hitachi 835–50, Tokyo, Japan).

### Real-time quantitative PCR (qRT-PCR) analysis

The total RNA and cDNA were prepared as described above. To quantify the expression levels of genes related to peroxisomal H_2_O_2_ production and IAA metabolism in leaves of various *GLO* transgenic rice lines and WT, qRT-PCR analysis was performed on a Bio-Rad CFX96 apparatus with SYBR Green I dye (Vazyme). PCR was carried out in 96-well plates using the following program: denaturation for 5 min at 95 °C, followed by 40 cycles of denaturation for 10 s at 95 °C and incorporative annealing and extension for 30 s at 60 °C. The primers used for qRT-PCR were designed on a dedicated website (https://biodb.swu.edu.cn/qprimerdb/?tds-ourcetag=s_pcqq_aiomsg). The data were normalized to the amplification of the *OsActin1* gene (Os03g0718100). All experiments were performed with three biological and three technical replicates per biological replicate. The primer sequences used in this paper are presented in Supporting Information (Additional files 9, 10).

## Supplementary Information

**Additional file 1.**

**Additional file 2.**

**Additional file 3.**

**Additional file 4.**

**Additional file 5.**

**Additional file 6.**

**Additional file 7.**

**Additional file 8.**

**Additional file 9.**

**Additional file 10.**

## Data Availability

All data generated or analyzed during this study are included in this published article and its supplementary information files; the datasets and accession numbers used during the current study (Os03g0786100, Os04g0623500, Os07g0152900, Os07g0616500, Os01g0108600, Os09g0385700, Os01g0732700, Os07g0437000, Os03g0790700, Os04g0118100, Os07g0675133, Os09g0544900, Os09g0133200, Os04g0653600, Os03g0313100, Os01g0783700, Os09g0527700, Os11g0490600, Os10g0147400, Os11g0169200, Os03g0718100) are available in the Rice Annotation Project (RAP) repository (https://rapdb.dna.affrc.go.jp/index.html).

## Abbreviations

GLO: Glycolate oxidase
CAT: Catalase
IBA: Indole-3-butyric acid
IAA: Indole-3-acetic acid
TSB: Tryptophan synthetase β
qRT-PCR: Real-time quantitative PCR
