# Supplementary material for: Glycolate oxidase-dependent H2O2 production regulates IAA biosynthesis in rice
Source: BMC Plant Biol. 2021 Jul 6;21:326. doi: 10.1186/s12870-021-03112-4 (PMC8261990; doi:10.1186/s12870-021-03112-4)
Supplement: Supplementary file 1 — Additional file 1. [file 12870_2021_3112_MOESM1_ESM.zip › Additional file 1.docx]

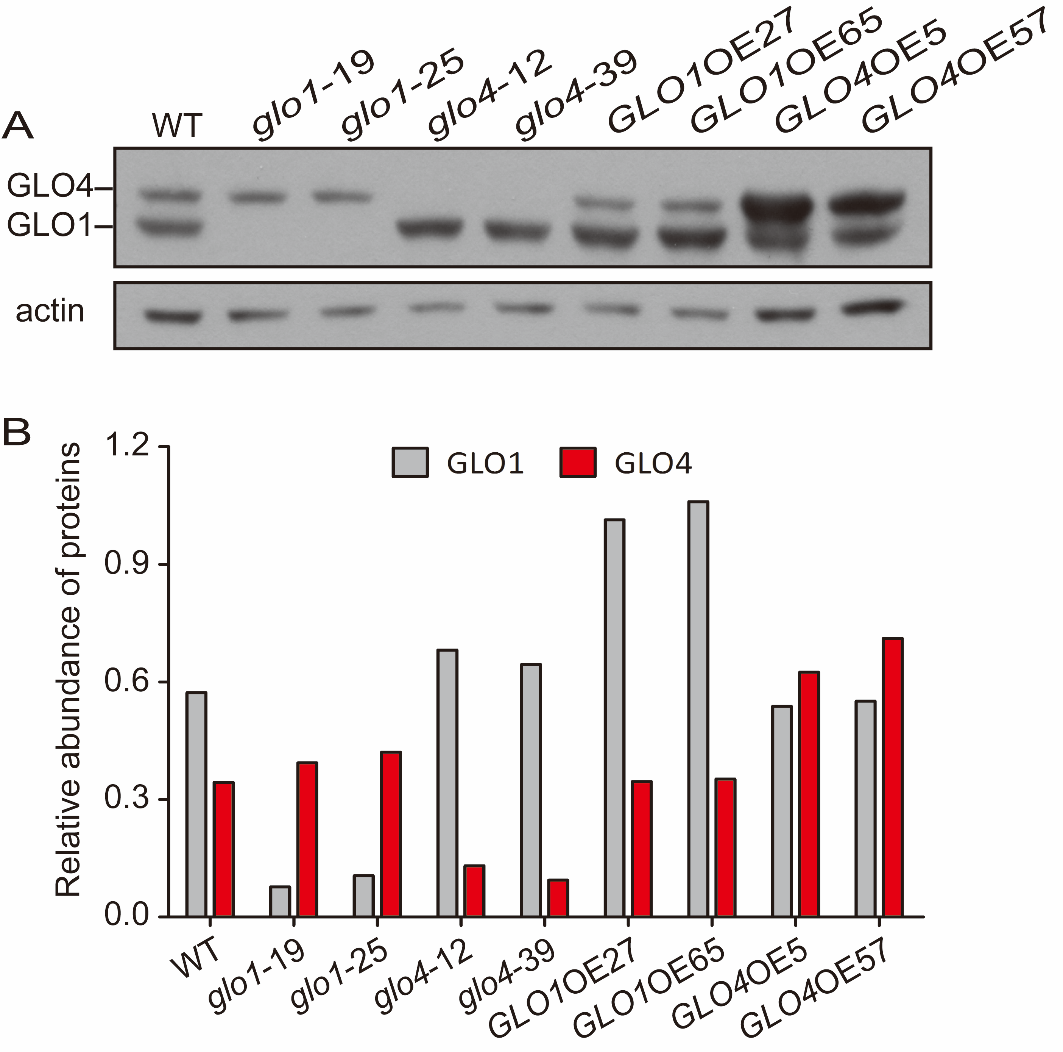


**Additional file 1** Western blotting analysis of each GLO isoform in different *GLO* genetically modified rice lines. Germinated seeds of *glo* mutants and *GLO* overexpression lines were cultured in Kimura B nutrient solution under natural conditions until five-leaf stage, and then leaves were detached for western blot detection. Western blot detection and calculation of relative GLO abundance using Image J in *glo1* and *glo4* mutants were shown in (A) and (B).
