# Supplementary figures and images for: Glycolate oxidase-dependent H2O2 production regulates IAA biosynthesis in rice
Source: BMC Plant Biol. 2021 Jul 6;21:326. doi: 10.1186/s12870-021-03112-4 (PMC8261990; doi:10.1186/s12870-021-03112-4)

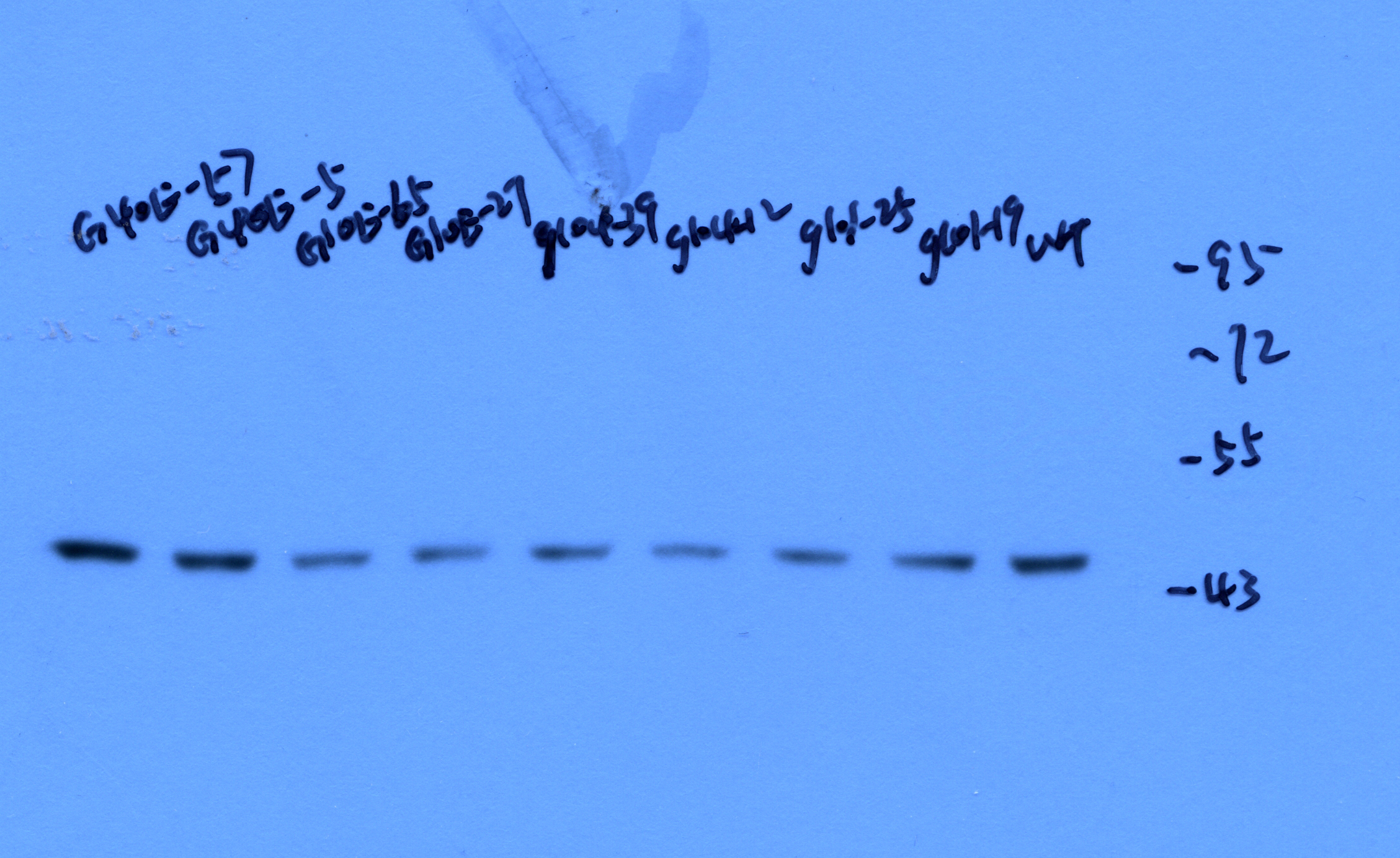

Supplement: Supplementary file 1 — Additional file 1. [file 12870_2021_3112_MOESM1_ESM.zip › Additional file 1A for actin.tif]

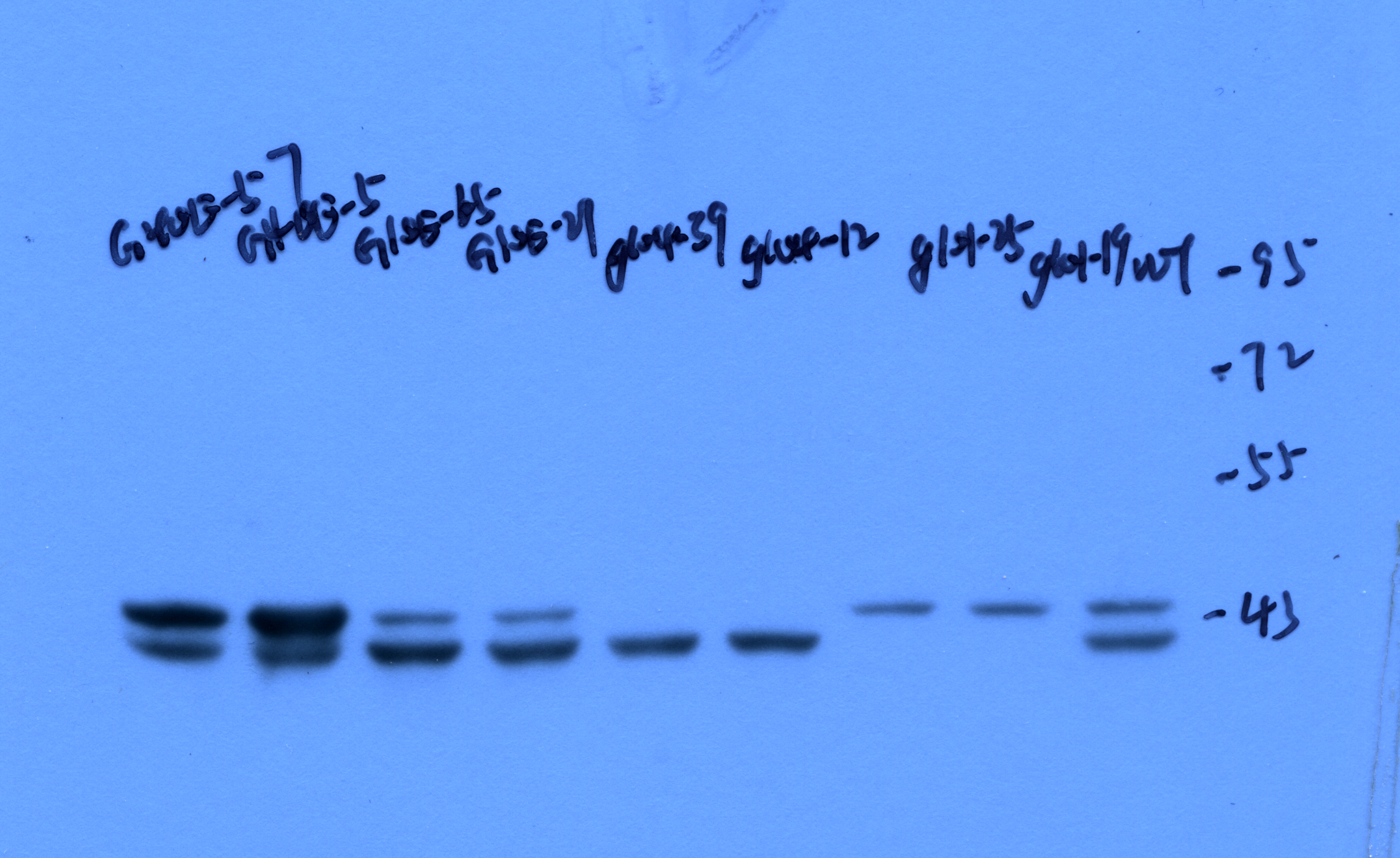

Supplement: Supplementary file 1 — Additional file 1. [file 12870_2021_3112_MOESM1_ESM.zip › Additional file 1A for GLO.tif]
