# Supplementary material for: Glycolate oxidase-dependent H2O2 production regulates IAA biosynthesis in rice
Source: BMC Plant Biol. 2021 Jul 6;21:326. doi: 10.1186/s12870-021-03112-4 (PMC8261990; doi:10.1186/s12870-021-03112-4)
Supplement: Supplementary file 2 — Additional file 2. [file 12870_2021_3112_MOESM2_ESM.docx]

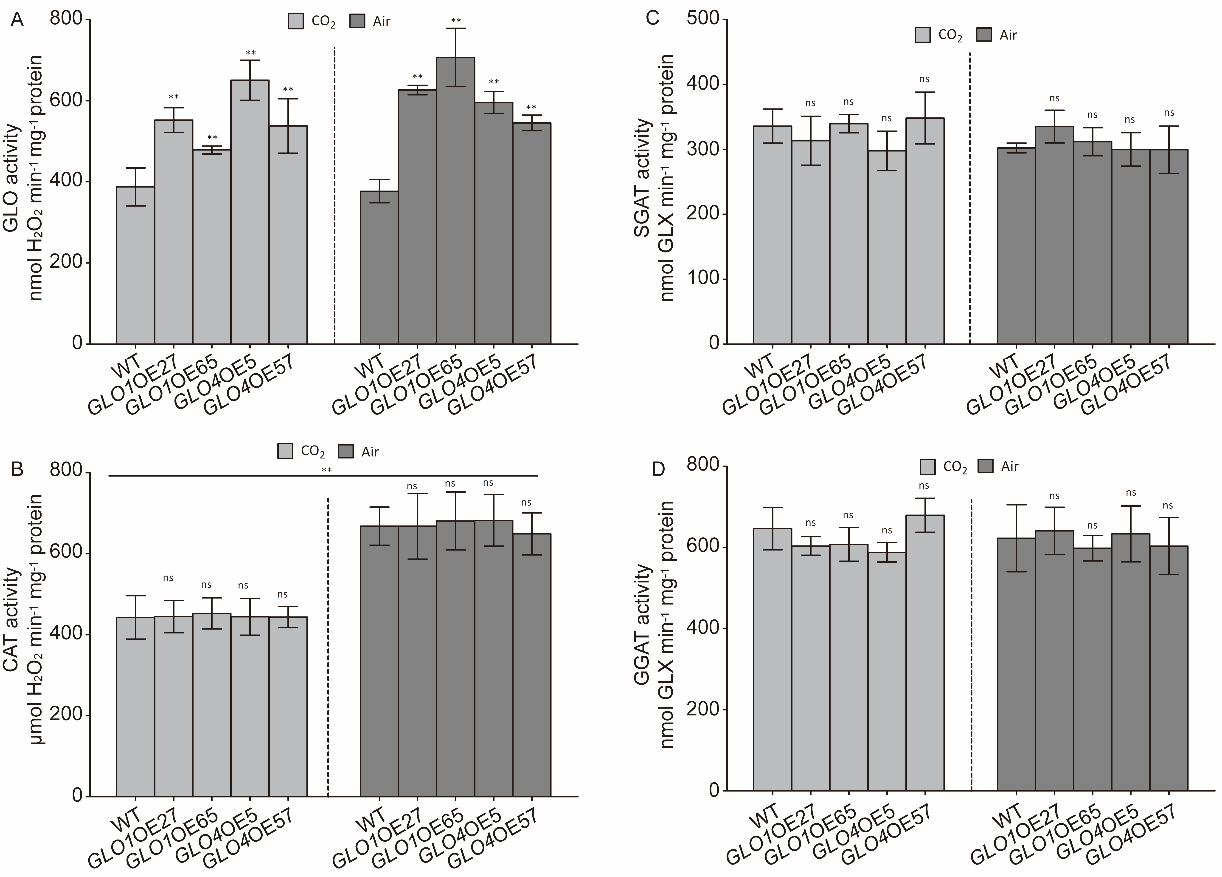


**Additional file 2** Activity assays of GLO, CAT, SGAT and GGAT in various *GLO* overexpression lines. Germinated seeds of different *GLO* overexpression lines were divided into two groups and cultured in two growth chambers under atmospheric and high CO_2_ (3500 ppm) conditions. The seedlings were then used for GLO (A), CAT (B), SGAT (C) and GGAT (D) activity measurement. Data are presented as means ± SD of three biological replications, *p < 0.05, **p < 0.01 according to Student’s *t*-test.
