# Supplementary material for: Glycolate oxidase-dependent H2O2 production regulates IAA biosynthesis in rice
Source: BMC Plant Biol. 2021 Jul 6;21:326. doi: 10.1186/s12870-021-03112-4 (PMC8261990; doi:10.1186/s12870-021-03112-4)
Supplement: Supplementary file 3 — Additional file 3. [file 12870_2021_3112_MOESM3_ESM.docx]

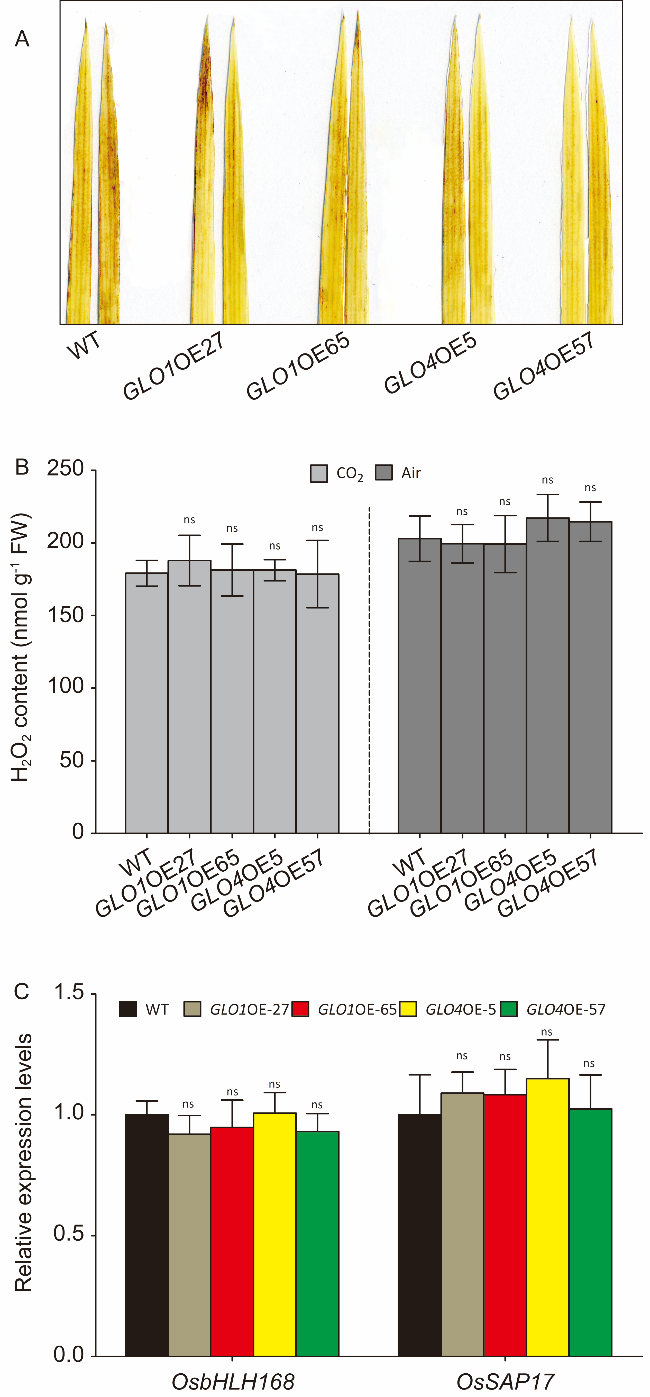


**Additional file 3** Detection of H_2_O_2_ level in various *GLO* overexpression lines. Germinated seeds of *GLO1*OE and *GLO4*OE were divided into two groups and cultured in two growth chambers under atmospheric and high CO_2_ (3500 ppm) conditions. H_2_O_2_-DAB staining of rice leaves under atmospheric condition (A) endogenous H_2_O_2_ contents of rice leaves under atmospheric condition and high CO_2_ condition (B); qRT-PCR analysis of peroxisomal H_2_O_2_-responsive genes in *glo1* and *glo4* mutants under atmospheric condition (C). The two H_2_O_2_ indicators selected were previously identified peroxisome-specific H_2_O_2_-responsive genes, *OsbHLH168* (Os01g0108600) and *OsSAP17* (Os09g0385700). Data are presented as means ± SD of three biological replications, *P < 0.05, **P < 0.01 according to Student’s *t*-tests.
