# Supplementary material for: Glycolate oxidase-dependent H2O2 production regulates IAA biosynthesis in rice
Source: BMC Plant Biol. 2021 Jul 6;21:326. doi: 10.1186/s12870-021-03112-4 (PMC8261990; doi:10.1186/s12870-021-03112-4)
Supplement: Supplementary file 4 — Additional file 4. [file 12870_2021_3112_MOESM4_ESM.docx]

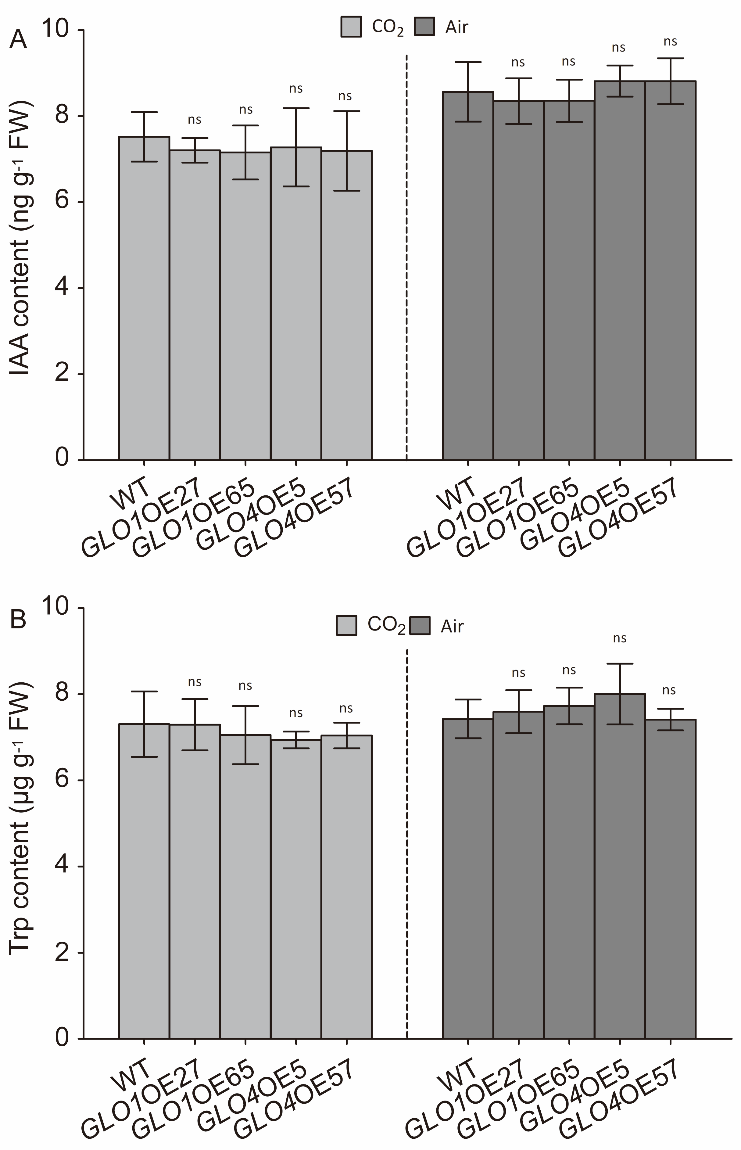


**Additional file 4** Changes of IAA and Trp levels in various *GLO* overexpression lines. Germinated seeds were cultured under atmospheric and high CO_2_ conditions. The leaves of five-leaf stage rice seedlings were then detached for determining IAA (A) and Trp (B). Data are presented as means ± SD of three biological replications, *P < 0.05, **P < 0.01 according to Student’s *t*-tests.
