# Supplementary material for: Glycolate oxidase-dependent H2O2 production regulates IAA biosynthesis in rice
Source: BMC Plant Biol. 2021 Jul 6;21:326. doi: 10.1186/s12870-021-03112-4 (PMC8261990; doi:10.1186/s12870-021-03112-4)
Supplement: Supplementary file 5 — Additional file 5. [file 12870_2021_3112_MOESM5_ESM.docx]

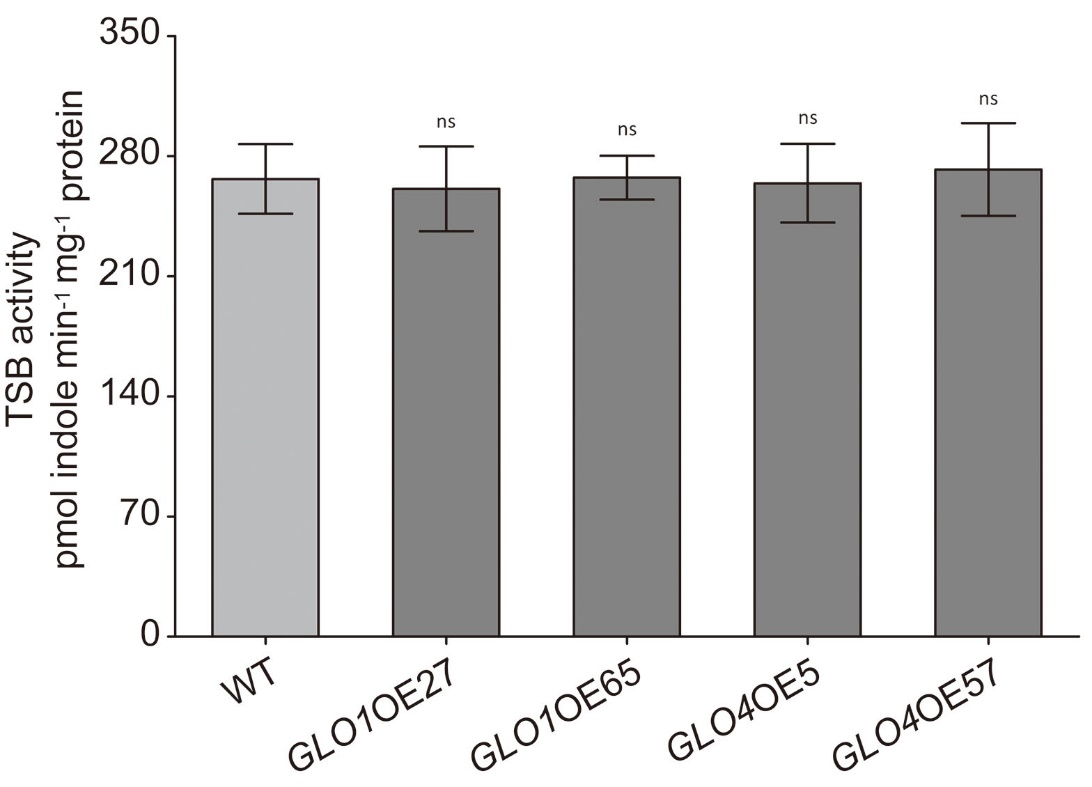


**Additional file 5** TSB activity in various *GLO* overexpression lines. The leaves of five-leaf stage rice seedlings grown in atmospheric condition were detached for measurement. Data are presented as means ± SD of three biological replications, *P < 0.05, **P < 0.01 according to Student’s *t*-tests.
