# Supplementary material for: Glycolate oxidase-dependent H2O2 production regulates IAA biosynthesis in rice
Source: BMC Plant Biol. 2021 Jul 6;21:326. doi: 10.1186/s12870-021-03112-4 (PMC8261990; doi:10.1186/s12870-021-03112-4)
Supplement: Supplementary file 6 — Additional file 6. [file 12870_2021_3112_MOESM6_ESM.docx]

**Additional file 6** Genes related to Trp-dependent IAA biosynthesis and conversion of IBA to IAA.

| **Locus names** | **Gene names** | **Functions** | **Refs** |
| --- | --- | --- | --- |
|  |  | **Trp-dependent IAA biosynthesis** |  |
| Os07g0182100 | *OsTSA1* | tryptophan synthase α subunit/the conversion of indoleglycerol phosphate to indole | Last et al*.*1991；Ribeiro et al*.*2016 |
| Os08g0135900 | *OsTSB1* | tryptophan synthase β subunit 1/the conversion of indole plus serine to tryptophan | Last et al*.* 1991；Li et al.2019 |
| Os01g0732700 | *OsYUC2* | indole-3-pyruvate monooxygenase YUCCA2/  the conversion of indole-3-pyruvate to IAA | Naser et al*.*2016 |
| Os07g0437000 | *OsYUC5* | indole-3-pyruvate monooxygenase YUCCA5/  the conversion of indole-3-pyruvate to IAA | Naser et al. 2016 |
| Os03g0790700 | *OsAO3* | aldehyde oxidase 3/the conversion of acetal-  dehyde (IAAld) to IAA | Abu-Zaitoon et al 2014 |
| Os04g0118100 | *OsAMI1* | amidase 1-like/participate in the formation of IAA via indole-3-acetaldoxime | Gao et al.2014；Godee et al*.*2017 |
|  |  | **The Conversion of IBA to IAA** |  |
| Os09g0133200 | *OsIBR1* | short-chain dehydrogenase/reductase | Zolman et al*.*2008 |
| Os07g0675133 | *OsIBR3* | acyl-CoA dehydrogenase/oxidase-like | Zolman et al. 2007 |
| Os09g0544900 | *OsECH2* | enoyl-CoA hydratase | Frick et al*.*2018；Strader et al*.*2011 |
