# Supplementary material for: Glycolate oxidase-dependent H2O2 production regulates IAA biosynthesis in rice
Source: BMC Plant Biol. 2021 Jul 6;21:326. doi: 10.1186/s12870-021-03112-4 (PMC8261990; doi:10.1186/s12870-021-03112-4)
Supplement: Supplementary file 7 — Additional file 7. [file 12870_2021_3112_MOESM7_ESM.docx]

**Additional file 7** IAA-responsive/transport genes and their functions.

| **Locus names** | **Gene names** | **Functions** | **Refs** |
| --- | --- | --- | --- |
| Os04g0653600 | *OsAIL5* | ethylene-responsive transcription factor | Horstman et al*.* 2014 |
| Os03g0313100 | *OsAIL7* | ethylene-responsive transcription factor | Nole-Wilson et al*.* 2005 |
| Os01g0783700 | *OsPBP1* | calcium-binding protein | Benjamins et al*.* 2003 |
| Os09g0527700 | *OsIAA26* | auxin-responsive protein | Du et al*.* 2013；Jiang et al*.* 2019；Jung et al*.* 2015 |
| *Os09g0437100* | *OsBGL* | serine, glycine and glutamine-rich protein | Wang et al*.* 2012；Mishra et al*.* 2017 |
| Os04g0662400 | *OsARG7* | indole-3-acetic acid-induced protein | Somyong et al*.* 2018 |
| Os11g0490600 | *OsLAZY1* | auxin polar transport/gravitropism | Chen et al*.* 2012；Yoshihara et al*.* 2017 |
| Os10g0147400 | *OsNS3* | auxin transporter-like protein | Xie et al*.* 2018 |
| Os11g0169200 | *OsNS4* | putative auxin transporter-like protein | Xie et al*.* 2018 |
