# Supplementary material for: Glycolate oxidase-dependent H2O2 production regulates IAA biosynthesis in rice
Source: BMC Plant Biol. 2021 Jul 6;21:326. doi: 10.1186/s12870-021-03112-4 (PMC8261990; doi:10.1186/s12870-021-03112-4)
Supplement: Supplementary file 8 — Additional file 8. [file 12870_2021_3112_MOESM8_ESM.docx]

**Additional file 8** Specific targeting sequences of *GLO1* and *GLO4* knockout lines edited by CRISPR/Cas9 systerm.

| **Gene name** | **Targets** | **specific targeting sequences** |
| --- | --- | --- |
| *OsGLO1* | 1 | CGCGCGTGCGCAGCGAGGC**TGG** |
|  | 2 | TTGAATTGCGTTTGCAGATC**GGG** |
| *OsGLO4* | 1 | CGGCGTCTTCGTGAGTACAC**CGG** |
|  | 2 | TTTGCCGGCCGACGGATGAT**CGG** |
