# Supplementary material for: Glycolate oxidase-dependent H2O2 production regulates IAA biosynthesis in rice
Source: BMC Plant Biol. 2021 Jul 6;21:326. doi: 10.1186/s12870-021-03112-4 (PMC8261990; doi:10.1186/s12870-021-03112-4)
Supplement: Supplementary file 9 — Additional file 9. [file 12870_2021_3112_MOESM9_ESM.docx]

**Additional file 9** Primer pairs of Trp-dependent IAA metabolism genes used in qRT-PCR analysis.

| **Gene name** | **qFprimer** | **qRprimer** |
| --- | --- | --- |
|  | **primers of genes related to IAA biosynthesis and conversion of IBA to IAA** | |
| *OsTSA1* | CTACACGTGCACTATCAAAAGG | TACAACAGTCATGAAGTTTGCG |
| *OsTSB1* | TTGAAAGGGTGCAATACAACAG | CTCCGCCAATAACATCATTCAG |
| *OsYUC2* | TGAAGACATACGATCGTCTCAG | CTGCTCATCATATTCTGCACAC |
| *OsYUC5* | CTCAAGGGAAGTGACTTCTTCA | TTGTGAAGCCAACAGAGTAGAG |
| *OsAO3* | CTCAACGCCTTCTGGAAGAAAG | CCTTATCTCCGACTTGAGGAAC |
| *OsAMI1* | GTTTCTGCAGAGAATGTCATCC | CACTTTGGTTACACGAGACAAC |
| *OsIBR1* | ATTGACAGGAGCACACTTAAGA | GCTGTAATGAATGATGCATCGT |
| *OsIBR3* | GTCTCTCATCAGACACAGTTCT | TTCGTTCAATAAAAACGGGGAC |
| *OsECH2* | ATCAAACAAAGGTCAAGGAACG | CATTTGAAGATAGGTGCAGAGC |
|  | **primers of IAA-responsive/transport genes** | |
| *OsAIL5* | CAGGGGAAAAGAGAGGTAATGT | CTACAAAAGGGTAAAAGGGCAG |
| *OsAIL7* | GAGGCCAAATCACTCTGATGAG | CAGGAGGTTGTGGATGAGATAC |
| *OsPBP1* | GCGTTCCAGTGTTGACATAATC | ATCCTCAAAACGCTCACTGTAT |
| *OsIAA26* | GTCTTCTTCGTCGAGGTCAG | CATGCTCACCTTCACGAAGTA |
| *OsBGL* | TGAGGTTAGGGGTAGAGTTGTA | TACAATGGTGGAGAGTACATGG |
| *OsARG7* | AGAGCCAAAGAGAGATCGAAAA | GAATGAGGCTGATCGAACGAT |
| *OsLAZY1* | TAAACCGGCTTCCAAGATGAAG | GTTGAACTCTTTGAACACGTCA |
| *OsNS3* | CGGCCTCTAGCTAATTAATCGA | GTAGCTCGCTAAAAACTAGTGC |
| *OsNS4* | TTGTGTCTGCTTAGGAGAGATG | TGTACAAACAGCAAGTTGATGG |
