# Supplementary material for: Glycolate oxidase-dependent H2O2 production regulates IAA biosynthesis in rice
Source: BMC Plant Biol. 2021 Jul 6;21:326. doi: 10.1186/s12870-021-03112-4 (PMC8261990; doi:10.1186/s12870-021-03112-4)
Supplement: Supplementary file 10 — Additional file 10. [file 12870_2021_3112_MOESM10_ESM.docx]

**Additional file 10** Primer pairs for peroxisomal H_2_O_2_-response genes used in qRT-PCR analysis.

| **Gene name** | **qFprimer** | **qRprimer** |
| --- | --- | --- |
| *OsbHLH168* | GCGACAAGATCTTCTACACTCT | AGAGTGAAAGAGTGGTGTGAAT |
| *OsSAP17* | CTCACGTTCTCCAACACGAG | GCGTGACAAGTCAGAAAATCTT |
